# The Structural Biology of Eastern Equine Encephalitis Virus, an Emerging Viral Threat

**DOI:** 10.3390/pathogens10080973

**Published:** 2021-07-31

**Authors:** S. Saif Hasan, Debajit Dey, Suruchi Singh, Matthew Martin

**Affiliations:** 1 Department of Biochemistry and Molecular Biology, University of Maryland School of Medicine, 108 N. Greene Street, Baltimore, MD 21201, USA; ddey@som.umaryland.edu (D.D.); suruchi.singh@som.umaryland.edu (S.S.); mfm.98.805@gmail.com (M.M.); 2 Center for Biomolecular Therapeutics, University of Maryland School of Medicine, 9600 Gudelsky Drive, Rockville, MD 20850, USA; 3 University of Maryland Marlene and Stewart Greenebaum Cancer Center, University of Maryland Medical Center, 22. S. Greene St., Baltimore, MD 21201, USA

**Keywords:** alphavirus, antibody, assembly, eastern equine encephalitis virus, structure

## Abstract

Alphaviruses are arboviruses that cause arthritis and encephalitis in humans. Eastern Equine Encephalitis Virus (EEEV) is a mosquito-transmitted alphavirus that is implicated in severe encephalitis in humans with high mortality. However, limited insights are available into the fundamental biology of EEEV and residue-level details of its interactions with host proteins. In recent years, outbreaks of EEEV have been reported mainly in the United States, raising concerns about public safety. This review article summarizes recent advances in the structural biology of EEEV based mainly on single-particle cryogenic electron microscopy (cryoEM) structures. Together with functional analyses of EEEV and related alphaviruses, these structural investigations provide clues to how EEEV interacts with host proteins, which may open avenues for the development of therapeutics.

## 1. Introduction

Alphaviruses are enveloped icosahedral arboviruses that infect mammalian hosts, including humans [1,2,3,4,5,6,7]. Alphaviruses are broadly classified as arthritogenic and encephalitic based on the disease associated with their infection. For instance, Chikungunya virus (CHIKV), an arthritogenic alphavirus, was responsible for an epidemic that affected millions in the Indian subcontinent and islands in the Indian Ocean in 2006 [8]. In contrast to arthritogenic alphaviruses, infections of encephalitic alphaviruses are often associated with mortality, especially for EEEV, which is endemic mostly to the Americas [9,10,11,12,13,14,15,16]. EEEV is transmitted by the mosquito *Culiseta melanura* mostly to birds [17,18,19]. Although EEEV typically causes fewer than ten reported human cases per year, 34 cases of EEEV were reported in USA in 2019 and the virus has been detected in mosquitoes that feed on humans [16,18,20]. EEEV infections have a high mortality rate of up to 75%, and nearly 90% of survivors report permanent neurological sequelae [21,22,23]. It has been reported that EEEV can be spread as an aerosol, which has led to its classification as a select agent by United States Department of Agriculture (USDA) and Centers for Disease Control and Prevention (CDC) [24,25]. At present, there are no approved vaccines or therapeutics to prevent or contain EEEV infections in the general human population [17,26,27]. Hence, EEEV represents an emerging threat to human health. However, molecular insights into EEEV infection and interactions of its proteins with host factors are rather limited as compared to other alphaviruses such as CHIKV. In this review article, we provide a summary of recent publications on the structural biology of EEEV entry, disassembly, assembly, and interactions with host molecules. We highlight how EEEV escapes detection by receptors for viral glycans and provide a perspective on antibody interactions with viral envelope proteins. The structural questions on EEEV dynamics in host cells raised in this article have broad relevance to the understanding of alphavirus infection and interactions with host cell molecules.

## 2. Overview of Alphavirus Infection and Assembly Cycles

**Figure 1:**
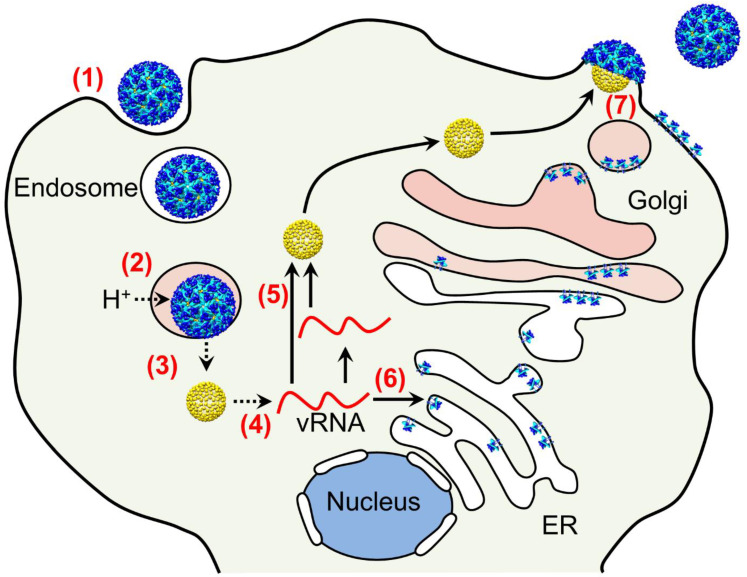
Entry and replication cycle of alphaviruses. Entry and disassembly steps are highlighted in red ((1) to (4)), whereas progeny virus assembly and exit are from step (5) to (7). (Step 1) Receptor-mediated endocytosis allows alphavirus particles to enter cells (particles shown in blue, cyan). (Step 2) Viral and endosomal membranes undergo fusion upon acidification of the endosome lumen (pink). (Step 3) Cytosolic release of the nucleocapsid core (yellow). (Step 4) Disintegration of the core and release of the viral RNA genome (vRNA, red line). (Step 5) Cytosolic progeny RNA genome synthesis and nucleocapsid core assembly occur in the cytosol, whereas envelope proteins are synthesized in secretory ER and Golgi network (Step 6). (Step 7) Final assembly of alphavirus progeny particles involves plasma membrane. Figure taken from [33].

Alphaviruses such as EEEV have a 12 kb positive-sense single-stranded RNA genome with five structural genes encoding capsid, E3, E2, 6K, Tf, and E1, and four non-structural genes encoding nsP1, nsP2, nsP3, and nsP4 [1,28,29,30]. The present review article focuses on the structural proteins capsid, E2, and E1 that constitute viral particles [28,31,32]. Alphaviruses have a complex infection cycle that is summarized in Figure 1 [33]. This involves viral entry into host cells, low pH triggered conformational changes and subsequent fusion with endosomal membrane, release of the nucleocapsid in the cytosol, disruption of the capsid shell to release the viral RNA genome, biosynthesis of progeny RNA and proteins, assembly of progeny nucleocapsid, and assembly and release of progeny alphavirus particles [1,34,35]. Hence, multiple host organelles are involved in the alphavirus infection and assembly cycles. These distinct steps present potential targets for therapeutic intervention.

## 3. SINV-EEEV Chimera and EEEV Virus-Like Particles (VLPs) for Structural Analyses

As a select agent, EEEV requires biological containment, which poses challenges for structural studies. Two reagents have been developed for structural studies of intact particles. First, a SINV-EEEV chimera has been described with SINV TR339 non-structural genes and RNA replication control elements and EEEV strain FL93-939 structural genes, i.e., for capsid, E3, E2, 6K, and E1 proteins [36,37]. These chimeric particles are replication competent and require biological safety level-2 containment. The structure of these particles has been determined to an average resolution of 4.4 Å by single-particle cryoEM [33]. Second, VLPs of EEEV have been developed by co-expression of genes for capsid, E3, E2, 6K, and E1 proteins from EEEV PE-6 [38]. These particles have been characterized by single-particle cryoEM to an average resolution of 4.2 Å [39]. Both SINV-EEEV chimera and EEEV VLPs have an overall icosahedral arrangement that is consistent with previously described cryoEM structures of alphaviruses [40,41,42,43,44,45,46,47,48,49].

## 4. E1 and E2 Glycoproteins: Tools to Penetrate Host Membranes

**Figure 2:**
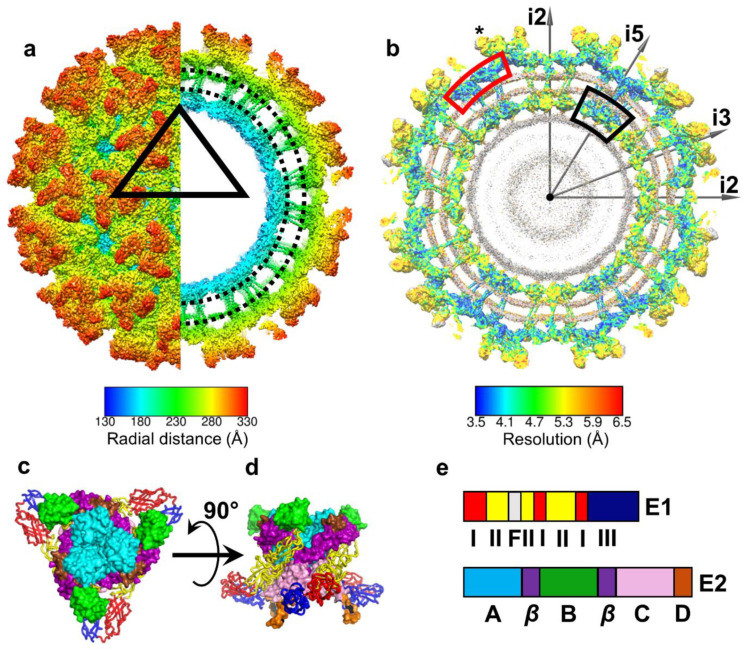
Structure of EEEV. (**a**) Surface representation of EEEV using radial coloring. An internal section of the cryoEM map is shown on the right (black dotted lines highlight lipid bilayer). (**b**) Resolution range and distribution in the cryoEM map highlighting prominent features such as envelope proteins and capsid protein (scale at bottom). The RNA genome (gray) was not included in this analysis. Gray arrows show directions of icosahedral symmetry axes for reference. Color code for boxes: red, E1 ecto-domain; black, capsid proteins in vicinity of icosahedral 5-fold axis; “*”, E2 ecto-domain. (**c**–**e**) Organization of E1–E2 trimeric spike ecto-domain shown in (**c**) in a radial orientation and (**d**) side-view rotated by 90° from (**c**). For clarity, E1 and E2 ecto-domains are shown in different representations as ribbon and surface, respectively. (**e**) Domain organization of E1 and E2 ecto-domains. The color codes as in (**c**,**d**). “F” represents the fusion loop. Figure taken from [33].

The alphavirus envelope consists of three proteins, E1, E2, and E3 [28,30,50,51]. Substantial insights into the structure and function of these proteins have been obtained from prior investigations on other alphaviruses, using both purified proteins and intact virus particles [52,53,54,55,56]. The E2 protein is implicated in receptor binding, whereas E1 is involved in low pH fusion with the host membrane [44,57,58,59,60,61,62]. This low pH-driven interaction of the alphavirus spike with host membranes raises an intriguing question, i.e., how is premature fusion inhibited during progeny spike biogenesis and assembly in the acidic Golgi lumen? The E3 protein has been shown to associate with the E1–E2 spike under acidic conditions to stabilize the hetero-dimer and to inhibit premature fusion [63]. Neutral pH in the extracellular medium drives dissociation of furin-cleaved E3 from the E1–E2 hetero-dimer [63,64,65,66,67]. The 4.4 Å resolution cryoEM structure of SINV-EEEV chimera identified the location of E1 and E2 proteins but not of E3 [33] (Figure 2a–e). Presumably, E3 was dissociated from the viral envelope during progeny assembly and purification. It is noted that E3 was reported in the cryoEM map of Venezuelan Equine Encephalitis Virus (VEEV) [48]. The SINV-EEEV cryoEM map corresponding to the E1–E2 ecto-domains has a resolution of approximately 3.5–6.0 Å (Figure 2a,b). The E1–E2 ecto-domains are organized in a hetero-dimer that closely resembles the hetero-dimer reported for SINV and CHIKV by X-ray crystallography [55,56]. The E1 and E2 ecto-domains are enriched in β-strands and demonstrate a three-domain organization, i.e., domains I, II, and III for E1 and A, B, and C for E2 (Figure 2c–e). Intra-chain disulfide bonds stabilize the respective ecto-domains of E1 and E2 proteins. Three E2 monomers in each trimeric spike demonstrate a radial arrangement, whereas three E1 monomers are organized tangentially, which is consistent with previous structural investigations (e.g., [41,48]).

**Figure 3:**
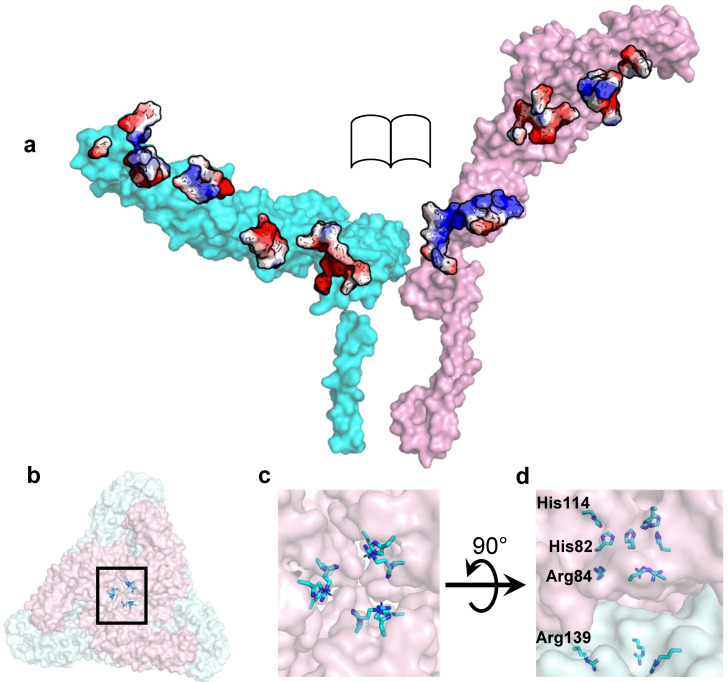
Electrostatic interactions in SINV-EEEV E1–E2. (**a**) Complementary charged surfaces in E1 (cyan) and E2 (pink) ecto-domain interface. Color code, acidic in red and basic in blue. (**b**–**d**) Basic residues shown as sticks in the trimeric E2–E2 interface (pink). (**b**) Trimeric spike along 3-fold axis. Black box highlights the basic interfacial residues, which are magnified in (**c**). (**d**) Four basic residues from one E2 ecto-domain are labelled. Figure taken from [33].

The envelope layer in alphaviruses undergoes pH-driven conformational changes [68,69,70]. This includes a rearrangement of tangential E1 ecto-domain into a radial orientation for insertion into the endosomal membrane and subsequent release of the alphavirus genome into cytosol [54,58,71,72]. The residue-level details of this membrane interaction are currently not well understood. The cryoEM analysis of SINV-EEEV particles generated new hypotheses relevant to these spike conformational changes [33]. For instance, the cryoEM structure of SINV-EEEV showed an E1–E2 interface that is highly enriched in charged, protonatable residues (Figure 3a) [33]. This suggests a possible pH-sensing mechanism for E1–E2 dissociation and envelope layer disassembly as suggested for related alphaviruses [73,74,75,76]. Such a hetero-dimer dissociation event would release the E1 ecto-domain for interactions with the endosomal membrane. This investigation proposed conservation in the mechanism of hetero-dimer dissociation as the likely pH-responsive residues in the E1–E2 electrostatic interface are widely conserved in alphaviruses [33]. Low pH initiated changes also involve a radial to tangential re-orientation of the E2 ecto-domain, which requires disruption of E2–E2 contacts proximal to the spike 3-fold axis [53,69,72]. This E2–E2 interface in SINV-EEEV is enriched in basic residues that demonstrate limited sequence conservation amongst alphaviruses (Figure 3b–d). This suggests the possibility that the pH sensitivity of the E2–E2 interface is alphavirus-specific. However, experimental validation of the above-mentioned hypotheses and residue-specific testing are still lacking. Nevertheless, the involvement of distinct early to late endosomal compartments hints towards distinct pH requirements for entry of different alphaviruses [77,78,79,80].

Flaviviruses, such as Dengue and Zika viruses, are similar to alphaviruses in their icosahedral symmetry and the presence of an internal membrane, although there is no sequence relationship between these two distinct genera [50,81]. Structural analyses have demonstrated similarities in the fold and organization of E1 protein in alphaviruses and envelope (E) protein in flaviviruses [54,82,83]. It has been noted that the acidic character of E1 (theoretical isoelectric point of ecto-domain, 6.0–6.7) is shared by the flavivirus E protein (theoretical isoelectric point of ecto-domain, 5.8–6.8), suggesting likely conserved low pH neutralization of acidic residues in membrane insertion [33].

## 5. A Multifunctional Capsid Protein

**Figure 4:**
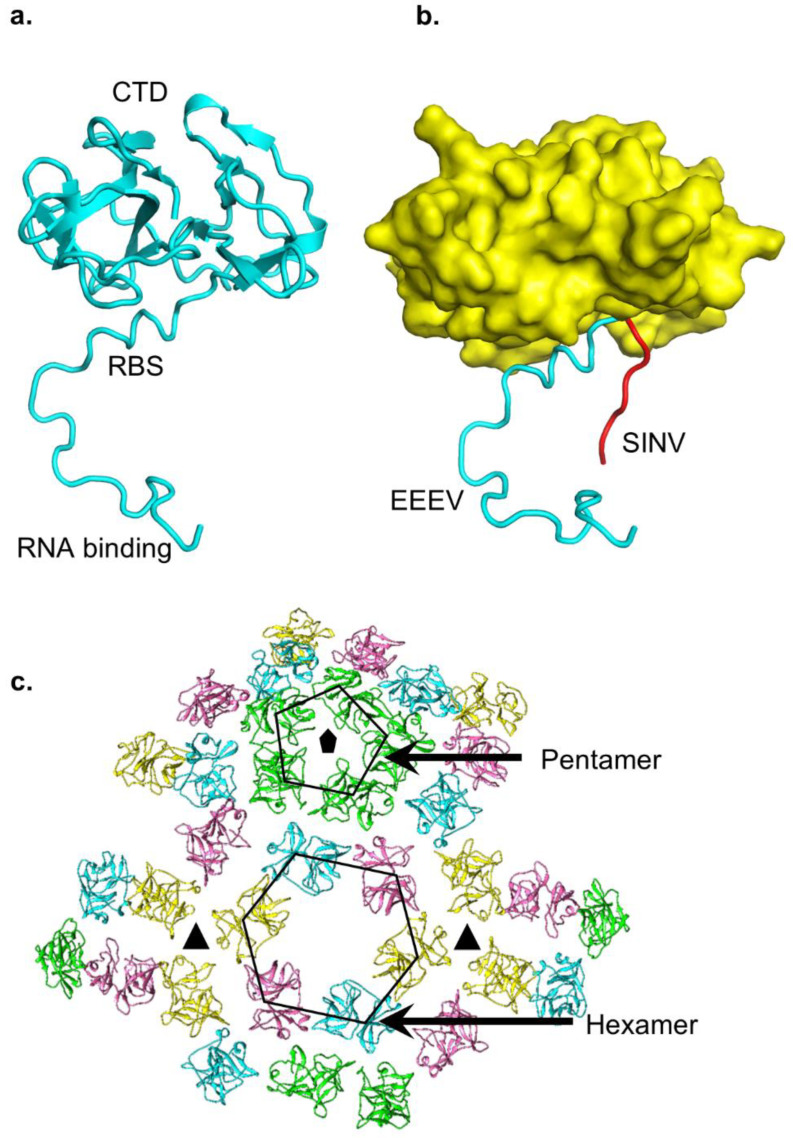
Structure of alphavirus capsid protein. (**a**) Capsid protein from SINV-EEEV. The RBS and NTD form an extended structure that is associated with the compact chymotrypsin-like CTD. (**b**) Structural superposition of SINV-EEEV and SINV capsid CTD (yellow surface) demonstrates difference in location of NTD residues between capsid of EEEV (cyan) and SINV (red). (**c**) In the internal capsid layer, pentamers and hexamers are arranged at the icosahedral 5- and 2-fold axes, respectively. These provide major stabilizing capsid–capsid contacts, whereas no interactions are observed near the 3-fold. In (**a**,**b**), coordinates for capsid protein (PDB ID 6MX7 for SINV-EEEV, 1SVP for SINV) were downloaded from PDB and figures were generated in PyMol (www.pymol.org, (accessed on 1 June 2021), version 1.8.6.0). Panel (**c**) taken from [33].

The capsid protein of SINV-EEEV demonstrates a two-domain organization characteristic of alphaviruses and is divided into an N-terminal (NTD, residues 1–116) and a C-terminal domain (CTD, residues 117–261) [33,48,52,84]. The alphavirus capsid NTD is enriched in basic residues that have been suggested to interact with the viral RNA genome [85,86]. A recent investigation has identified multiple sites for capsid protein binding on the viral genomic RNA [87]. The capsid NTD in EEEV demonstrates an abundance of Gly and Pro residues, which are characteristic of intrinsically disordered proteins (reviewed in [88]). This is consistent with cryoEM reconstructions of intact alphavirus particles wherein a large segment of the capsid NTD is not visualized (e.g., [48]). It is inferred that this is due to disorder in the capsid NTD. However, cryoEM reconstructions of alphaviruses utilize icosahedral symmetry for averaging between 60 asymmetric units. This raises the question of whether there is symmetry mismatch between the outer layer of envelope proteins and inner capsid NTD, which would contribute to relatively poor reconstruction of the capsid NTD. This possibility has not been explored in detail. Nevertheless, it has been postulated that the intrinsic disorder of the capsid NTD may provide structural flexibility for interactions with dissimilar sequences in the viral RNA genome [33]. This is because the icosahedral protein shell of alphaviruses encloses a single copy of the viral RNA genome. The alphavirus genome sequence associated with each of the capsid proteins will be unique, leading to dissimilar genome–capsid interactions at these sites. Flexibility in the capsid NTD may facilitate these interactions while minimizing the genetic cost of encoding specialized sequences for each capsid position. Insights into the organization of the capsid NTD are derived from a comparison of a crystal structure of the SINV capsid protein and the cryoEM structures of SINV-EEEV and related VEEV [33,48,52,85]. Coordinates for short segments of the capsid NTD attached to the CTD have been refined in these maps. However, the coordinates of these short segments of capsid NTD show considerable differences (Figure 4a,b). For instance, in the SINV crystal structure, the NTD segment displays an elongated conformation that extends “radially” from the CTD [85]. An analysis of crystal packing shows that this short segment is involved in contacts with adjacent symmetry-related molecules [85]. In contrast, a comparable segment of the capsid NTD in the SINV-EEEV cryoEM structure has a more tangential organization, although the map is at a lower resolution [33]. However, in both structures the NTD short segment shows an extended conformation wherein the side-chains of the basic residues are available for interactions with the acidic viral RNA genome. The extended conformation of SINV-EEEV capsid NTD is consistent with a comparable, but shorter capsid NTD sequence mapped in the VEEV cryoEM structure [33,48]. Despite these advances in elucidating the structure of the capsid NTD, limited insights are available into the stoichiometry of genome-capsid interactions within an alphavirus particle, whether all 240 capsid protein copies bind the genome, and whether there is local “order” at the capsid–genome interaction site that is not visualized by current icosahedral averaging approaches. Addressing these questions is essential for a deeper understanding of genome packaging and alphavirus assembly.

The alphavirus capsid CTD has been characterized in substantially more detail than NTD. Functionally, the CTD encodes a protease activity that is essential for alphavirus polyprotein processing and is consistent with a chymotrypsin protease-like fold of CTD [52,85]. The CTD contains a mixed secondary structure content of short α-helices and β-strands. A groove in the capsid CTD provides the interaction surface for the C-terminal residues of the E2 glycoprotein [33,48,89,90]. In the cryoEM structure of SINV-EEEV, this CTD groove is enriched in polar interactions from side chains of Arg132, Tyr159, Tyr177, Trp244, and Thr250. Hydrophobic and Van der Waals interactions are provided by CTD Phe134 and Ile158. Mutagenesis of SINV capsid CTD residues in this groove has implicated Tyr162, which is equivalent to EEEV capsid Tyr159, and Lys252 in alphavirus assembly [89].

The capsid protein in alphaviruses provides a protective shell around the viral genomic RNA [50]. An analysis of the SINV-EEEV and other alphavirus cryoEM structures shows differences in interactions between adjacent capsid CTD with respect to the icosahedral symmetry axes (Figure 4c) [33,48,50]. For instance, neighboring capsid CTDs proximal to the icosahedral 2-fold and 5-fold vertices are organized into hexagonal and pentagonal facets. Three capsid CTDs near an icosahedral 3-fold vertex belong to three adjacent capsid hexagons. These three capsid CTDs are more widely separated and do not demonstrate inter-capsid interactions, unlike the capsid pentagons and hexagons [33,50]. This suggests that stability in the nucleocapsid core is provided by CTD linkage with the E2 layer, which is closely packed with extensive inter-protein contacts [42,51,91]. In this model, disruption of envelope–capsid interactions upon alphavirus entry and low pH-triggered fusion releases the nucleocapsid core into the cytosol, devoid of stabilizing E2 contacts, followed by subsequent core disassembly. Although the experimental validity of this model of nucleocapsid disassembly remains to be tested, it is consistent with prior investigations that suggest nucleocapsid disassembly in the host cell cytosol follows alphavirus entry [92,93,94,95]. These structural data on EEEV reinforce several interesting questions that are yet to be fully addressed. For instance, if the nucleocapsid core is inherently unstable, then how is it assembled during progeny assembly in the cytosol prior to interactions with the E2 glycoprotein? Is the nucleocapsid core assembled initially in a metastable state(s)? Investigations of in vitro-assembled cores in alphaviruses such as RRV and WEEV have provided clues to this assembly process wherein the envelope-free cores maintain an overall arrangement similar to that inside viral particles [96,97]. In complementary structural analyses, icosahedral symmetry was demonstrated in newly assembled VEEV cores, although the diameter was substantially larger than in intact virus particles [98,99,100]. Furthermore, the isolation of envelope-free nucleocapsid cores by detergent treatment and gradient purification has been reported [101]. These data suggest that the cores are assembled in a somewhat stable, icosahedral state prior to complete alphavirus assembly. However, more recent data present a contrasting picture wherein a lack of nucleocapsid uniformity is noted even in fully assembled alphavirus particles [97]. These results suggest that while the envelope layer provides protection to the core, association with the envelope layer may be insufficient to generate an ideal capsid icosahedron. Presently, it is not known what causes this deviation in nucleocapsid core symmetry, the extent to which these differences in structural uniformity are important for assembly/disassembly, and if this non-uniformity is a widely conserved feature in alphaviruses. Recent data on genome-less capsid cores add another layer of complexity to the role of the viral genome in driving nucleocapsid assembly [102,103]. Overall, the understanding of uncoating [104], symmetry, organization, assembly of alphavirus nucleocapsid cores is limited despite recent advances. Progress in cryoEM imaging inside cells and improvement in resolution of such structures should help address several of the unanswered questions pertaining to alphavirus nucleocapsid cores.

## 6. Structural Basis of Heparan Sulfate (HS) Binding in EEEV 

**Figure 5:**
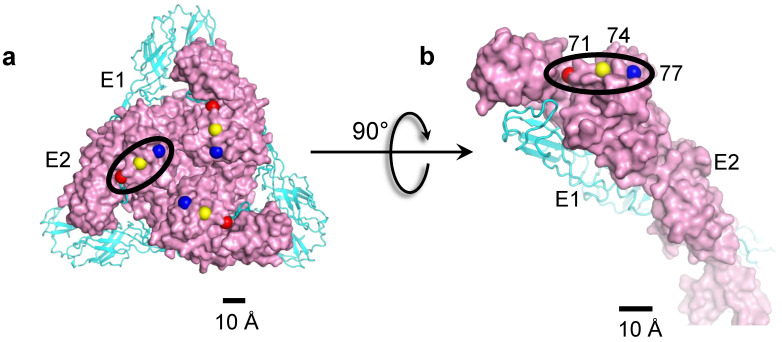
HS binding residues in E2 of SINV-EEEV. (**a**) The Cα-atoms of HS binding residues (Lys71, red; Lys74, yellow; Lys77, blue) shown as spheres, whereas the E2 ectodomain is shown in a surface representation (pink). (**b**) A rotated side-view shows one linear triad highlighted in a black oval. Figure taken from [33].

HS is a long anionic polysaccharide polymer linked to plasma membrane proteins and is involved in a variety of functions, such as cellular adhesion, signaling, and coagulation (reviewed in [105,106]). Cell culture adaptation of alphaviruses is associated with an HS binding phenotype and decreased pathogenesis [59,60,107,108,109,110]. However, in EEEV, an HS binding phenotype is associated with host infection [111,112]. The sequencing of North American strains of EEEV host isolates identified key basic residues in the E2 ecto-domain (Lys71, Lys74, and Lys77) associated with HS binding. Of these three basic residues, Lys74 of E2 is widely conserved, Lys77 is least conserved, and residue Lys71 is semi-conserved and often replaced by His, which is also basic [33,111,112]. The 4.4 Å cryoEM structure of SINV-EEEV chimera placed these residues in a “linear triad” in E2 (Figure 5a,b) [33]. In this linear triad, access to the Lys71 side-chain is partly occluded by domain B, whereas Lys74 and Lys77 are well exposed. This linear triad is surrounded by a predominantly electrostatic protein environment. Hence, the putative binding site for HS is distributed over three adjacent symmetry-related E2 monomers.

Structural insights into the interactions of the SINV-EEEV with HS were obtained from single-particle cryoEM analysis of SINV-EEEV particles complexed with heparin (Hp), a low-molecular-weight HS analog [113]. SINV-EEEV demonstrates specific interactions with Hp, which reduces infection of SINV-EEEV chimera in cell culture by almost 90% upon pre-incubation with Hp. In contrast, the sulfated polysaccharide polymer chondroitin sulfate (CS), which is similar to HS, only reduces infection by 30%, suggesting stereo-specificity in SINV-EEEV for Hp and HS. The cryoEM structure of SINV-EEEV in the presence of Hp yielded surprising insights. No features corresponding to Hp were observed in the vicinity of the linear triad of Lys71–Lys74–Lys77. It was proposed that this could be due to a non-icosahedral arrangement of Hp, which likely results in a loss of features during reconstruction. Alternatively, this implies that the linear Lys triad is not directly involved in binding Hp, but is rather selective for endogenous host HS, which is more complex and larger [114]. In a trimeric spike, linear Lys triads from three monomers in a quaternary organization would provide multiple interactions with high avidity for HS, in contrast to a single linear Lys triad from one spike monomer. The substantially smaller chain length of Hp in the cryoEM analysis could have been insufficient to satisfy this complex interaction with multiple linear Lys triads. Hence, even though Hp molecules were not visualized by cryoEM analysis of SINV-EEEV, a role of the linear Lys triad in HS binding prior to viral entry cannot be excluded.

The cryoEM analysis of SINV-EEEV revealed four distinct sites for Hp binding that are unique with respect to icosahedral symmetry axes [113]. Two axial Hp sites overlap with the 3-fold rotational symmetry axis in q3 and i3 trimeric spikes, whereas two peripheral sites are in proximity to the E2 β-connector. The quaternary arrangement of residues in the axial sites provides multiple interactions for Hp binding. These axial sites are enriched in basic residues, whose side chains face the 3-fold rotational symmetry axes, and the bound Hp molecules. In the axial as well as peripheral sites, the bound Hp molecules appear to be spherical, which is likely a consequence of multiple binding poses and 3-fold averaging in the i3 spikes. Hence, this cryoEM analysis suggests that HS interactions in EEEV involve multiple sites in the viral E2 protein, which could provide multiple routes towards neurovirulence [111,112].

## 7. Glycosylation of E1 and E2 Proteins in EEEV

**Figure 6:**
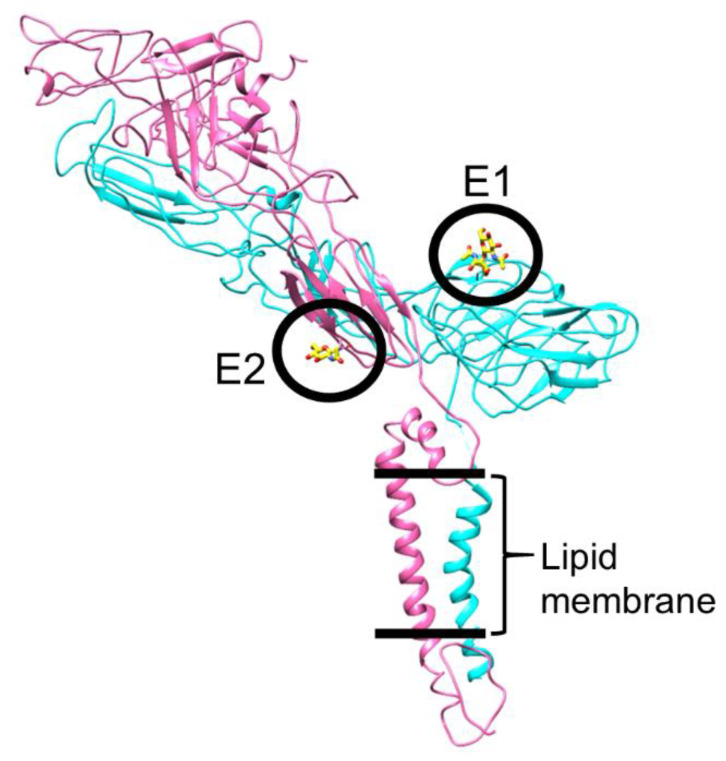
Glycosylation in EEEV E1-E2 proteins. The glycan groups are highlighted as sticks in yellow-red and are encircled for clarity. Color code, E1, cyan, E2, pink. Figure taken from [33].

Post-translational modifications such as glycosylation play a key role in alphavirus infection and assembly cycles. Mutations in the alphavirus envelope protein glycosylation sites affect infectivity, membrane fusion, assembly, and yield of progeny virus [91,115,116,117,118,119]. Elimination of E2 glycosylation sites enhances viral interaction with HS [118]. E2 glycosylation interacts with host cell DC-SIGN and L-SIGN molecules for viral entry [120]. DC-SIGN/L-SIGN are lectin molecules in macrophage and dendritic cell plasma membranes that demonstrate strong interactions with pathogen high-mannose glycosylation sites to activate downstream immune responses [121,122,123,124,125,126,127]. The structural analysis of SINV-EEEV chimera generated insights into the location and function of glycosylation sites (Figure 6) [33]. A sequence analysis showed one N-linked glycosylation site each on E1 and E2 ecto-domains of EEEV, i.e., E1 Asn134 and E2 Asn315. These two sites conform to the well-established glycosylation motif, Asn-X-Thr (X = any residue except Pro), and have the sequence Asn134-Ile135-Thr136 and Asn315-Phe316-Thr317 for E1 and E2, respectively. The cryoEM analysis of SINV-EEEV particles was consistent with this sequence-based prediction. The cryoEM map demonstrated the presence of a feature connected to the Asn side-chain in both E1 and E2. One N-acetyl-glucosamine monosaccharide was fitted in the E2 site, whereas a disaccharide was accommodated in the E1 site. The E2 glycosylation site is not accessible on the viral surface, whereas the E1 site is well-exposed near the 2-fold and 5-fold symmetry axes, close to the base of the trimeric spike. In the context of E2, this presents a contrast as other alphaviruses demonstrate at least one exposed glycosylation site on the E2 ecto-domain [91,128,129]. In the case of EEEV, this lack of an accessible E2 glycosylation site may provide a biological route to escape immune detection via DC-SIGN/L-SIGN [33]. A comparative analysis of infectivity was performed between EEEV and SINV, which was previously shown to interact efficiently with DC-SIGN and L-SIGN [120]. It was observed that EEEV demonstrates more limited infectivity in this cellular system than SINV, thus implicating the poorly exposed E2 glycan in inefficient interactions with host lectins. This also suggests that the E1 glycan is not sufficient for viral entry even though it is surface exposed. This is consistent with the observation that glycan composition is a key determinant of viral tropism [130]. A chemical analysis of the E1 glycan in purified SINV-EEEV particles showed enrichment in pauci-mannose carbohydrates in the virus from mosquito C6/36 cell lines and in complex-type carbohydrates in the virus from mammalian BHK-15 cells [33]. Oligo-mannose glycans, which are essential for interactions with DC-SIGN and L-SIGN, constituted only one-tenth and one-sixth of the total glycans in E1 in the mosquito- and mammalian-derived SINV-EEEV particles, respectively. Hence, the E1 glycan is biochemically sub-optimal for interactions with DC-SIGN/L-SIGN even though it is exposed on the viral surface [131]. It would be informative to test if the EEEV envelope glycans are essential for efficient viral protein biogenesis. If this hypothesis is confirmed, it would suggest that EEEV has likely evolved a means for envelope protein biogenesis while avoiding a deleterious side-effect of glycosylation, i.e., immune detection.

## 8. Structural Basis of EEEV Neutralization by Monoclonal Antibodies

The structural and functional basis of neutralization by monoclonal antibodies (mAbs) has been previously investigated for several alphaviruses such as arthritogenic CHIKV, RRV and MAYV, and encephalitic VEEV and WEEV [44,45,132,133,134,135,136,137,138,139,140,141,142,143,144,145,146,147,148,149,150]. In the context of EEEV, prior investigations have focused on characterization of immune response to E2 peptides and compared their cross-reactivity to VEEV [151,152,153,154]. Two recent cryoEM investigations have provided structural insights into the neutralization of EEEV by Fab fragments from neutralizing mAbs [33,39].

**Figure 7:**
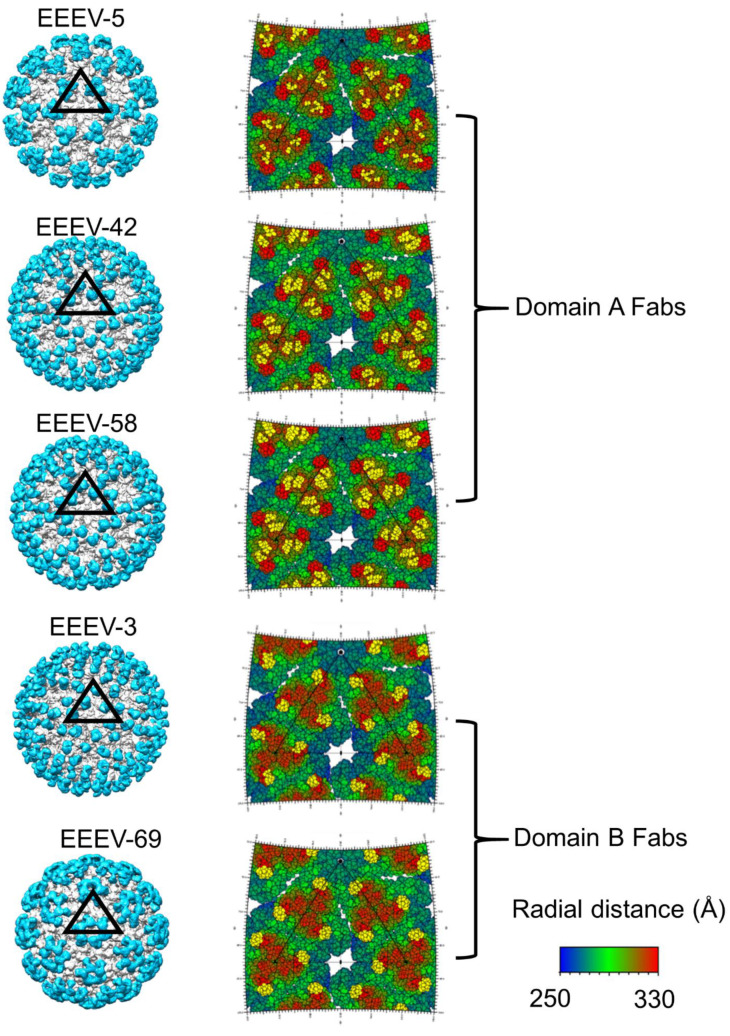
CryoEM structures of Fabs in complex with SINV-EEEV chimeric particles. The footprints of the Fabs are highlighted in yellow on the trimeric spike. Figure taken from [33].

**Figure 8:**
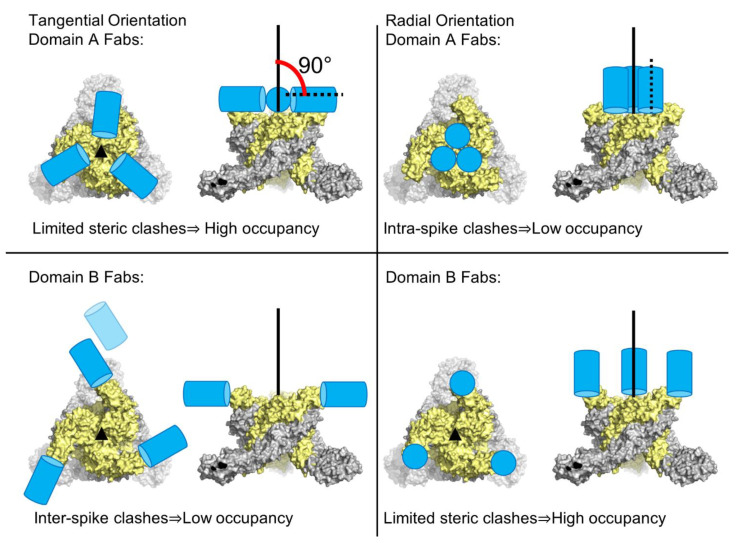
The role of steric limitations on Fab occupancy in alphaviruses. Color code: E1-E2, gray-yellow; Fab, blue; Fab quasi-2-fold axis, black dotted line. Tangential binding allows higher occupancy of domain A Fabs (upper left) unlike radial binding that is limited by clashes (upper right). In domain B Fabs, tangential binding is limited by clashes between Fabs bound to neighboring spikes and potentially neighboring E1–E2 proteins (lower left). High occupancy in domain B Fabs is favored in radial binding (lower right). Figure taken from [33].

In the first investigation [33,36], Fabs from five potent neutralizing mouse mAbs (EEEV-3, EEEV-5, EEEV-42, EEEV-58, and EEEV-69) were characterized in complex with SINV-EEEV chimera by single-particle cryoEM. These structures were determined to a resolution of 7.3–8.2 Å and all five Fabs were found to bind to the E2 ecto-domain (Figure 7). These five Fabs were classified into two groups based on their footprints, i.e., domain A Fabs (EEEV-5, EEEV-42, and EEEV-58) and domain B Fabs (EEEV-3, and EEEV-69). Even though each respective group of Fabs demonstrated substantially overlapping footprints, large differences in average occupancies were reported. For instance, EEEV-5 had a relative occupancy of 45.4% with respect to the ecto-domain, whereas EEEV-58 had a relative occupancy of 97.2%. Both Fabs were incubated in excess with the SINV-EEEV particles prior to flash-freezing for cryoEM analysis. This raises an intriguing question about the factors that contribute to this difference in Fab occupancies. It was suggested that the angular orientation of the Fab may play a role in its occupancy (Figure 8). In a trimeric spike, three symmetry-related copies of E2 domain A are clustered close there near the 3-fold rotational axis. Hence, a Fab that is bound in a radial orientation on domain A, and hence parallel to the spike 3-fold axis, would face steric clash from its symmetry-related partners. This would limit its average occupancy on the spike. However, domain A Fabs that deviate from this radial orientation and are more tangential would experience fewer steric restrictions. Hence, a tangential orientation of Fabs will be favored for high occupancy in domain A. Domain B Fabs present a contrasting scenario. Domain B of the E2 ecto-domain is located at the distal end of the trimeric spike, at a larger distance from the spike 3-fold axis than domain A. However, domain B from a trimeric spike is closer to domain B from an adjacent spike. Hence, a Fab bound tangentially to domain B could potentially pose steric restrictions on the binding of a second Fab to domain B from an adjacent trimeric spike. However, Fabs that demonstrate a more radial orientation are likely to face fewer steric restrictions. Based on this analysis, it was suggested that the more radial orientation of domain A Fab, EEEV-5, i.e., 14.5° with respect to the spike 3-fold axis, was partly responsible for this low 45.4% occupancy. Although this analysis was performed on SINV-EEEV, the conservation of E1–E2 organization and structure in alphaviruses suggests that this is widely applicable to investigations of steric limitations in Fab-alphavirus interactions.

A more recent structural analysis of SINV-EEEV with Fabs from two potent neutralizing human mAbs, EEEV-33 and EEEV-143, has provided insights into conformational epitopes [39]. These cryoEM structures showed that EEEV-33 is a domain A Fab, whereas EEEV-143 is a domain B Fab. This analysis demonstrated a radial orientation of EEEV-33 Fabs on the trimeric spike, which generates potential steric restrictions for binding of a divalent IgG. Here, it should be noted that Fabs from these two human mAbs possess strong neutralization activity, unlike monovalent Fabs from mouse mAbs with anti-EEEV neutralization activity. This suggests a likely interaction wherein the Fab from these human mAbs either sterically blocks access to the host membrane or cross-links the EEEV envelope. A binding analysis showed that EEEV-33 preferentially interacts with intact particles over purified E2 protein, indicating the involvement of a quaternary binding site. Hence, cross-linking of multiple adjacent subunits represents a potent mechanism for alphavirus neutralization.

It is likely that cross-linking of adjacent subunits by Fabs is a high probability event in alphaviruses from a structural perspective. In an alphavirus spike, the most highly exposed surfaces are of individual E1–E2 hetero-dimers. The likely sites on the alphavirus surface where adjacent subunits are in close contact are in proximity of the icosahedral 2, 3, and 5-fold axes. This is consistent with the cross-linking activity noted for EEEV-33, which recognizes a quaternary epitope near the spike 3-fold axis and in a cryoEM structure of CHIKV complexed with a bivalent IgG [155]. Overall, cross-linking of adjacent protein subunits is expected to be more efficient if the viral surface were “smoother”, wherein neighboring subunits are available for simultaneous binding by Fabs or mAbs. An example is mature flaviviruses whose envelope protein is arranged in a smooth, herringbone pattern [81,156,157,158,159,160,161]. In these enveloped icosahedral viruses, multiple sites for subunit cross-linking are accessible. This is noted by a relatively larger abundance of Fabs and mAbs whose epitopes are located close to the subunit–subunit interface (reviewed in [162,163]).

## 9. Conclusions

The investigations of SINV-EEEV chimeric particles and EEEV VLPs described above have greatly advanced the understanding of the structural basis of EEEV–host interactions, especially in the context of entry and antibody neutralization, while setting the stage for further investigations. For instance, it is not known which protein receptors in the host membrane are hijacked during EEEV entry. What are the intermediate states of E1–E2 ecto-domains during pH-triggered transitions in internalized EEEV, and more broadly in alphaviruses? Are these E1–E2 transition states conserved in alphaviruses and can they be exploited in the design of broad-spectrum inhibitors? Does association with host receptors affect E1–E2 conformational changes and disassembly? When Fabs and mAbs are provided in sub-stoichiometric quantities, what are the preferred epitope sites for Fab and mAbs binding on the crowded viral surface in EEEV and other alphaviruses? Is the binding of Fabs or mAbs at a few selected sites sufficient for neutralization? Addressing these questions will require a combination of cellular and functional approaches that build on high-resolution information derived from structural studies to gain further insights into alphavirus–host interactions.

## References

[1] Strauss J.H., Strauss E.G. (1994). The alphaviruses: Gene expression, replication, and evolution. Microbiol. Rev..

[2] Suhrbier A., Jaffar-Bandjee M.C., Gasque P. (2012). Arthritogenic alphaviruses—An overview. Nat. Rev. Rheumatol..

[3] Levi L.I., Vignuzzi M. (2019). Arthritogenic alphaviruses: A worldwide emerging threat?. Microorganisms.

[4] Lwande O.W., Obanda V., Bucht G., Mosomtai G., Otieno V., Ahlm C., Evander M. (2015). Global emergence of alphaviruses that cause arthritis in humans. Infect. Ecol. Epidemiol..

[5] Rulli N.E., Melton J., Wilmes A., Ewart G., Mahalingam S. (2007). The molecular and cellular aspects of arthritis due to alphavirus infections: Lesson learned from Ross River virus. Ann. N. Y. Acad. Sci..

[6] Zacks M.A., Paessler S. (2010). Encephalitic alphaviruses. Vet. Microbiol..

[7] Giltner L.T., Shahan M.S. (1933). The immunological relationship of Eastern and Western strains of Equine encephalomyelitis virus. Science.

[8] WHO (2006). Outbreak news. Chikungunya and dengue, south-west Indian Ocean. Wkly. Epidemiol. Rec..

[9] Armstrong P.M., Andreadis T.G. (2013). Eastern equine encephalitis virus-Old enemy, new threat. N. Engl. J. Med..

[10] Ayres J.C., Feemster R.F. (1949). The sequelae of Eastern equine encephalomyelitis. N. Engl. J. Med..

[11] Lindsey N.P., Staples J.E., Fischer M. (2018). Eastern equine encephalitis virus in the United States, 2003–2016. Am. J. Trop. Med. Hyg..

[12] Lindsey N.P., Martin S.W., Staples J.E., Fischer M. (2020). Notes from the field: Multistate outbreak of Eastern equine encephalitis virus-United States, 2019. MMWR Morb. Mortal. Wkly. Rep..

[13] Andreadis T.G., Anderson J.F., Tirrell-Peck S.J. (1998). Multiple isolations of Eastern equine encephalitis and highlands J viruses from mosquitoes (Diptera: Culicidae) during a 1996 epizootic in southeastern Connecticut. J. Med. Entomol..

[14] Oliver J., Tan Y., Haight J.D., Tober K.J., Gall W.K., Zink S.D., Kramer L.D., Campbell S.R., Howard J.J., Das S.R. (2020). Spatial and temporal expansions of Eastern equine encephalitis virus and phylogenetic groups isolated from mosquitoes and mammalian cases in New York State from 2013 to 2019. Emerg. Microbes Infect..

[15] Silverman M.A., Misasi J., Smole S., Feldman H.A., Cohen A.B., Santagata S., McManus M., Ahmed A.A. (2013). Eastern equine encephalitis in children, Massachusetts and New Hampshire, USA, 1970–2010. Emerg. Infect. Dis..

[16] CDC Eastern Equine Encephalitis (EEE). https://www.cdc.gov/easternequineencephalitis/tech/epi.html.

[17] Morens D.M., Folkers G.K., Fauci A.S. (2019). Eastern equine encephalitis virus-Another emergent arbovirus in the United States. N. Engl. J. Med..

[18] Sherwood J.A., Stehman S.V., Howard J.J., Oliver J. (2020). Cases of Eastern equine encephalitis in humans associated with Aedes canadensis, Coquillettidia perturbans and Culiseta melanura mosquitoes with the virus in New York State from 1971 to 2012 by analysis of aggregated published data. Epidemiol. Infect..

[19] Weaver S.C., Scott T.W., Lorenz L.H., Repik P.M. (1991). Detection of Eastern equine encephalomyelitis virus deposition in Culiseta melanura following ingestion of radiolabeled virus in blood meals. Am. J. Trop. Med. Hyg..

[20] Mitchell C.J., Niebylski M.L., Smith G.C., Karabatsos N., Martin D., Mutebi J.P., Craig G.B., Mahler M.J. (1992). Isolation of Eastern equine encephalitis virus from *Aedes albopictus* in Florida. Science.

[21] Ronca S.E., Dineley K.T., Paessler S. (2016). Neurological sequelae resulting from encephalitic alphavirus infection. Front. Microbiol..

[22] Reddy A.J., Woods C.W., Welty-Wolf K.E. (2008). Eastern equine encephalitis leading to multi-organ failure and sepsis. J. Clin. Virol..

[23] Carrera J.P., Forrester N., Wang E., Vittor A.Y., Haddow A.D., Lopez-Verges S., Abadia I., Castano E., Sosa N., Baez C. (2013). Eastern equine encephalitis in Latin America. N. Engl. J. Med..

[24] Sidwell R.W., Smee D.F. (2003). Viruses of the Bunya- and Togaviridae families: Potential as bioterrorism agents and means of control. Antiviral Res..

[25] CDC-USDA HHS and USDA Select Agents and Toxins. https://www.selectagents.gov/sat/list.htm.

[26] Reichert E., Clase A., Bacetty A., Larsen J. (2009). Alphavirus antiviral drug development: Scientific gap analysis and prospective research areas. Biosecur. Bioterror.

[27] Trobaugh D.W., Sun C., Dunn M.D., Reed D.S., Klimstra W.B. (2019). Rational design of a live-attenuated Eastern equine encephalitis virus vaccine through informed mutation of virulence determinants. PLoS Pathog..

[28] Strauss E.G., Rice C.M., Strauss J.H. (1984). Complete nucleotide sequence of the genomic RNA of Sindbis virus. Virology.

[29] Faragher S.G., Meek A.D., Rice C.M., Dalgarno L. (1988). Genome sequences of a mouse-avirulent and a mouse-virulent strain of Ross River virus. Virology.

[30] Garoff H., Frischauf A.M., Simons K., Lehrach H., Delius H. (1980). Nucleotide sequence of cDNA coding for Semliki Forest virus membrane glycoproteins. Nature.

[31] Chang G.J., Trent D.W. (1987). Nucleotide sequence of the genome region encoding the 26S mRNA of Eastern equine encephalomyelitis virus and the deduced amino acid sequence of the viral structural proteins. J. Gen. Virol..

[32] J H Strauss A., Strauss E.G. (1988). Evolution of RNA viruses. Annu. Rev. Microbiol..

[33] Hasan S.S., Sun C., Kim A.S., Watanabe Y., Chen C.L., Klose T., Buda G., Crispin M., Diamond M.S., Klimstra W.B. (2018). Cryo-EM structures of Eastern equine encephalitis virus reveal mechanisms of virus disassembly and antibody neutralization. Cell Rep..

[34] Leung J.Y., Ng M.M., Chu J.J. (2011). Replication of alphaviruses: A review on the entry process of alphaviruses into cells. Adv. Virol..

[35] Jose J., Snyder J.E., Kuhn R.J. (2009). A structural and functional perspective of alphavirus replication and assembly. Future Microbiol..

[36] Kim A.S., Austin S.K., Gardner C.L., Zuiani A., Reed D.S., Trobaugh D.W., Sun C., Basore K., Williamson L.E., Crowe J.E. (2019). Protective antibodies against Eastern equine encephalitis virus bind to epitopes in domains A and B of the E2 glycoprotein. Nat. Microbiol..

[37] Zhang R., Kim A.S., Fox J.M., Nair S., Basore K., Klimstra W.B., Rimkunas R., Fong R.H., Lin H., Poddar S. (2018). Mxra8 is a receptor for multiple arthritogenic alphaviruses. Nature.

[38] Ko S.Y., Akahata W., Yang E.S., Kong W.P., Burke C.W., Honnold S.P., Nichols D.K., Huang Y.S., Schieber G.L., Carlton K. (2019). A virus-like particle vaccine prevents equine encephalitis virus infection in nonhuman primates. Sci. Transl. Med..

[39] Williamson L.E., Gilliland T., Yadav P.K., Binshtein E., Bombardi R., Kose N., Nargi R.S., Sutton R.E., Durie C.L., Armstrong E. (2020). Human antibodies protect against aerosolized Eastern equine encephalitis virus infection. Cell.

[40] Kostyuchenko V.A., Jakana J., Liu X., Haddow A.D., Aung M., Weaver S.C., Chiu W., Lok S.M. (2011). The structure of Barmah Forest virus as revealed by cryo-electron microscopy at a 6Å resolution has detailed transmembrane protein architecture and interactions. J. Virol..

[41] Mancini E.J., Clarke M., Gowen B.E., Rutten T., Fuller S.D. (2000). Cryo-electron microscopy reveals the functional organization of an enveloped virus, Semliki Forest virus. Mol. Cell.

[42] Mukhopadhyay S., Zhang W., Gabler S., Chipman P.R., Strauss E.G., Strauss J.H., Baker T.S., Kuhn R.J., Rossmann M.G. (2006). Mapping the structure and function of the E1 and E2 glycoproteins in alphaviruses. Structure.

[43] Sherman M.B., Weaver S.C. (2010). Structure of the recombinant alphavirus Western equine encephalitis virus revealed by cryoelectron microscopy. J. Virol..

[44] Smith T.J., Cheng R.H., Olson N.H., Peterson P., Chase E., Kuhn R.J., Baker T.S. (1995). Putative receptor binding sites on alphaviruses as visualized by cryoelectron microscopy. Proc. Natl. Acad. Sci. USA.

[45] Sun S., Xiang Y., Akahata W., Holdaway H., Pal P., Zhang X., Diamond M.S., Nabel G.J., Rossmann M.G. (2013). Structural analyses at pseudo atomic resolution of Chikungunya virus and antibodies show mechanisms of neutralization. eLife.

[46] Zhang W., Mukhopadhyay S., Pletnev S.V., Baker T.S., Kuhn R.J., Rossmann M.G. (2002). Placement of the structural proteins in Sindbis virus. J. Virol..

[47] Zhang W., Heil M., Kuhn R.J., Baker T.S. (2005). Heparin binding sites on Ross River virus revealed by electron cryo-microscopy. Virology.

[48] Zhang R., Hryc C.F., Cong Y., Liu X., Jakana J., Gorchakov R., Baker M.L., Weaver S.C., Chiu W. (2011). 4.4 Å cryo-EM structure of an enveloped alphavirus Venezuelan equine encephalitis virus. EMBO J..

[49] Ribeiro-Filho H.V., Coimbra L.D., Cassago A., Rocha R.P.F., Guerra J., de Felicio R., Carnieli C.M., Leme L., Padilha A.C., Paes Leme A.F. (2021). Cryo-EM structure of the mature and infective Mayaro virus at 4.4 Å resolution reveals features of arthritogenic alphaviruses. Nat. Commun..

[50] Cheng R.H., Kuhn R.J., Olson N.H., Rossmann M.G., Choi H.K., Smith T.J., Baker T.S. (1995). Nucleocapsid and glycoprotein organization in an enveloped virus. Cell.

[51] Forsell K., Xing L., Kozlovska T., Cheng R.H., Garoff H. (2000). Membrane proteins organize a symmetrical virus. EMBO J..

[52] Choi H.K., Tong L., Minor W., Dumas P., Boege U., Rossmann M.G., Wengler G. (1991). Structure of Sindbis virus core protein reveals a chymotrypsin-like serine proteinase and the organization of the virion. Nature.

[53] Gibbons D.L., Vaney M.C., Roussel A., Vigouroux A., Reilly B., Lepault J., Kielian M., Rey F.A. (2004). Conformational change and protein-protein interactions of the fusion protein of Semliki Forest virus. Nature.

[54] Lescar J., Roussel A., Wien M.W., Navaza J., Fuller S.D., Wengler G., Wengler G., Rey F.A. (2001). The fusion glycoprotein shell of Semliki Forest virus: An icosahedral assembly primed for fusogenic activation at endosomal pH. Cell.

[55] Li L., Jose J., Xiang Y., Kuhn R.J., Rossmann M.G. (2010). Structural changes of envelope proteins during alphavirus fusion. Nature.

[56] Voss J.E., Vaney M.C., Duquerroy S., Vonrhein C., Girard-Blanc C., Crublet E., Thompson A., Bricogne G., Rey F.A. (2010). Glycoprotein organization of Chikungunya virus particles revealed by X-ray crystallography. Nature.

[57] Haag L., Garoff H., Xing L., Hammar L., Kan S.T., Cheng R.H. (2002). Acid-induced movements in the glycoprotein shell of an alphavirus turn the spikes into membrane fusion mode. EMBO J..

[58] Wahlberg J.M., Bron R., Wilschut J., Garoff H. (1992). Membrane fusion of Semliki Forest virus involves homotrimers of the fusion protein. J. Virol..

[59] Byrnes A.P., Griffin D.E. (1998). Binding of Sindbis virus to cell surface heparan sulfate. J. Virol..

[60] Klimstra W.B., Ryman K.D., Johnston R.E. (1998). Adaptation of Sindbis virus to BHK cells selects for use of heparan sulfate as an attachment receptor. J. Virol..

[61] Omar A., Koblet H. (1988). Semliki Forest virus particles containing only the E1 envelope glycoprotein are infectious and can induce cell-cell fusion. Virology.

[62] Wahlberg J.M., Garoff H. (1992). Membrane fusion process of Semliki Forest virus. I: Low pH-induced rearrangement in spike protein quaternary structure precedes virus penetration into cells. J. Cell Biol..

[63] Sjoberg M., Lindqvist B., Garoff H. (2011). Activation of the alphavirus spike protein is suppressed by bound E3. J. Virol..

[64] Bonatti S., Blobel G. (1979). Absence of a cleavable signal sequence in Sindbis virus glycoprotein PE2. J. Biol. Chem..

[65] Bonatti S., Migliaccio G., Blobel G., Walter P. (1984). Role of signal recognition particle in the membrane assembly of Sindbis viral glycoproteins. Eur. J. Biochem..

[66] Firth A.E., Chung B.Y., Fleeton M.N., Atkins J.F. (2008). Discovery of frameshifting in alphavirus 6K resolves a 20-year enigma. Virol. J..

[67] Liljestrom P., Garoff H. (1991). Internally located cleavable signal sequences direct the formation of Semliki Forest virus membrane proteins from a polyprotein precursor. J. Virol..

[68] Wu S.R., Haag L., Hammar L., Wu B., Garoff H., Xing L., Murata K., Cheng R.H. (2007). The dynamic envelope of a fusion class II virus. Prefusion stages of Semliki Forest virus revealed by electron cryomicroscopy. J. Biol. Chem..

[69] Cao S., Zhang W. (2013). Characterization of an early-stage fusion intermediate of Sindbis virus using cryoelectron microscopy. Proc. Natl. Acad. Sci. USA.

[70] Paredes A.M., Ferreira D., Horton M., Saad A., Tsuruta H., Johnston R., Klimstra W., Ryman K., Hernandez R., Chiu W. (2004). Conformational changes in Sindbis virions resulting from exposure to low pH and interactions with cells suggest that cell penetration may occur at the cell surface in the absence of membrane fusion. Virology.

[71] Klimjack M.R., Jeffrey S., Kielian M. (1994). Membrane and protein interactions of a soluble form of the Semliki Forest virus fusion protein. J. Virol..

[72] Gibbons D.L., Erk I., Reilly B., Navaza J., Kielian M., Rey F.A., Lepault J. (2003). Visualization of the target-membrane-inserted fusion protein of Semliki Forest virus by combined electron microscopy and crystallography. Cell.

[73] Fields W., Kielian M. (2013). A key interaction between the alphavirus envelope proteins responsible for initial dimer dissociation during fusion. J. Virol..

[74] Qin Z.L., Zheng Y., Kielian M. (2009). Role of conserved histidine residues in the low-pH dependence of the Semliki Forest virus fusion protein. J. Virol..

[75] Sahoo B., Gudigamolla N.K., Chowdary T.K. (2020). Acidic pH-induced conformational changes in Chikungunya virus fusion protein E1: A spring-twisted region in the domain I–III linker acts as a hinge point for swiveling motion of domains. J. Virol..

[76] Zheng Y., Sanchez-San Martin C., Qin Z.L., Kielian M. (2011). The domain I-domain III linker plays an important role in the fusogenic conformational change of the alphavirus membrane fusion protein. J. Virol..

[77] van Duijl-Richter M.K., Hoornweg T.E., Rodenhuis-Zybert I.A., Smit J.M. (2015). Early events in Chikungunya virus infection-From virus cell binding to membrane fusion. Viruses.

[78] Colpitts T.M., Moore A.C., Kolokoltsov A.A., Davey R.A. (2007). Venezuelan equine encephalitis virus infection of mosquito cells requires acidification as well as mosquito homologs of the endocytic proteins Rab5 and Rab7. Virology.

[79] Marsh M., Bolzau E., Helenius A. (1983). Penetration of Semliki Forest virus from acidic prelysosomal vacuoles. Cell.

[80] Kolokoltsov A.A., Fleming E.H., Davey R.A. (2006). Venezuelan equine encephalitis virus entry mechanism requires late endosome formation and resists cell membrane cholesterol depletion. Virology.

[81] Kuhn R.J., Zhang W., Rossmann M.G., Pletnev S.V., Corver J., Lenches E., Jones C.T., Mukhopadhyay S., Chipman P.R., Strauss E.G. (2002). Structure of dengue virus: Implications for flavivirus organization, maturation, and fusion. Cell.

[82] Rey F.A., Heinz F.X., Mandl C., Kunz C., Harrison S.C. (1995). The envelope glycoprotein from tick-borne encephalitis virus at 2 Å resolution. Nature.

[83] Roussel A., Lescar J., Vaney M.C., Wengler G., Wengler G., Rey F.A. (2006). Structure and interactions at the viral surface of the envelope protein E1 of Semliki Forest virus. Structure.

[84] Linger B.R., Kunovska L., Kuhn R.J., Golden B.L. (2004). Sindbis virus nucleocapsid assembly: RNA folding promotes capsid protein dimerization. RNA.

[85] Lee S., Owen K.E., Choi H.K., Lee H., Lu G., Wengler G., Brown D.T., Rossmann M.G., Kuhn R.J. (1996). Identification of a protein binding site on the surface of the alphavirus nucleocapsid and its implication in virus assembly. Structure.

[86] Owen K.E., Kuhn R.J. (1996). Identification of a region in the Sindbis virus nucleocapsid protein that is involved in specificity of RNA encapsidation. J. Virol..

[87] Brown R.S., Anastasakis D.G., Hafner M., Kielian M. (2020). Multiple capsid protein binding sites mediate selective packaging of the alphavirus genomic RNA. Nat. Commun..

[88] Uversky V.N. (2013). A decade and a half of protein intrinsic disorder: Biology still waits for physics. Protein. Sci..

[89] Tang J., Jose J., Chipman P., Zhang W., Kuhn R.J., Baker T.S. (2011). Molecular links between the E2 envelope glycoprotein and nucleocapsid core in Sindbis virus. J. Mol. Biol..

[90] Chen L., Wang M., Zhu D., Sun Z., Ma J., Wang J., Kong L., Wang S., Liu Z., Wei L. (2018). Implication for alphavirus host-cell entry and assembly indicated by a 3.5Å resolution cryo-EM structure. Nat. Commun..

[91] Pletnev S.V., Zhang W., Mukhopadhyay S., Fisher B.R., Hernandez R., Brown D.T., Baker T.S., Rossmann M.G., Kuhn R.J. (2001). Locations of carbohydrate sites on alphavirus glycoproteins show that E1 forms an icosahedral scaffold. Cell.

[92] Wengler G., Wurkner D., Wengler G. (1992). Identification of a sequence element in the alphavirus core protein which mediates interaction of cores with ribosomes and the disassembly of cores. Virology.

[93] Wengler G., Gros C., Wengler G. (1996). Analyses of the role of structural changes in the regulation of uncoating and assembly of alphavirus cores. Virology.

[94] Wengler G. (2009). The regulation of disassembly of alphavirus cores. Arch. Virol..

[95] Wengler G., Wengler G. (1984). Identification of a transfer of viral core protein to cellular ribosomes during the early stages of alphavirus infection. Virology.

[96] Tellinghuisen T.L., Hamburger A.E., Fisher B.R., Ostendorp R., Kuhn R.J. (1999). In Vitro assembly of alphavirus cores by using nucleocapsid protein expressed in *Escherichia coli*. J. Virol..

[97] Wang J.C., Chen C., Rayaprolu V., Mukhopadhyay S., Zlotnick A. (2015). Self-assembly of an alphavirus core-like particle is distinguished by strong inter-subunit association energy and structural defects. ACS Nano.

[98] Lamb K., Lokesh G.L., Sherman M., Watowich S. (2010). Structure of a Venezuelan equine encephalitis virus assembly intermediate isolated from infected cells. Virology.

[99] Paredes A., Alwell-Warda K., Weaver S.C., Chiu W., Watowich S.J. (2003). Structure of isolated nucleocapsids from Venezuelan equine encephalitis virus and implications for assembly and disassembly of enveloped virus. J. Virol..

[100] Mukhopadhyay S., Chipman P.R., Hong E.M., Kuhn R.J., Rossmann M.G. (2002). In Vitro-assembled alphavirus core-like particles maintain a structure similar to that of nucleocapsid cores in mature virus. J. Virol..

[101] Enzmann P.J., Weiland F. (1980). Separation of alphavirus nucleocapsids from envelope fragments. Z. Naturforsch C Biosci..

[102] Button J.M., Mukhopadhyay S. (2020). Removing the polyanionic cargo requirement for assembly of alphavirus core-like particles to make an empty alphavirus core. Viruses.

[103] Rayaprolu V., Moore A., Wang J.C., Goh B.C., Perilla J.R., Zlotnick A., Mukhopadhyay S. (2017). Length of encapsidated cargo impacts stability and structure of in vitro assembled alphavirus core-like particles. J. Phys. Condens. Matter..

[104] Singh I., Helenius A. (1992). Role of ribosomes in Semliki Forest virus nucleocapsid uncoating. J. Virol..

[105] Esko J.D., Lindahl U. (2001). Molecular diversity of heparan sulfate. J. Clin. Investig..

[106] Jackson R.L., Busch S.J., Cardin A.D. (1991). Glycosaminoglycans: Molecular properties, protein interactions, and role in physiological processes. Physiol. Rev..

[107] Ryman K.D., Gardner C.L., Burke C.W., Meier K.C., Thompson J.M., Klimstra W.B. (2007). Heparan sulfate binding can contribute to the neurovirulence of neuroadapted and nonneuroadapted Sindbis viruses. J. Virol..

[108] Bernard K.A., Klimstra W.B., Johnston R.E. (2000). Mutations in the E2 glycoprotein of Venezuelan equine encephalitis virus confer heparan sulfate interaction, low morbidity, and rapid clearance from blood of mice. Virology.

[109] Heil M.L., Albee A., Strauss J.H., Kuhn R.J. (2001). An amino acid substitution in the coding region of the E2 glycoprotein adapts Ross River virus to utilize heparan sulfate as an attachment moiety. J. Virol..

[110] Smit J.M., Waarts B.L., Kimata K., Klimstra W.B., Bittman R., Wilschut J. (2002). Adaptation of alphaviruses to heparan sulfate: Interaction of Sindbis and Semliki forest viruses with liposomes containing lipid-conjugated heparin. J. Virol..

[111] Gardner C.L., Ebel G.D., Ryman K.D., Klimstra W.B. (2011). Heparan sulfate binding by natural Eastern equine encephalitis viruses promotes neurovirulence. Proc. Natl. Acad. Sci. USA.

[112] Gardner C.L., Choi-Nurvitadhi J., Sun C., Bayer A., Hritz J., Ryman K.D., Klimstra W.B. (2013). Natural variation in the heparan sulfate binding domain of the Eastern equine encephalitis virus E2 glycoprotein alters interactions with cell surfaces and virulence in mice. J. Virol..

[113] Chen C.L., Hasan S.S., Klose T., Sun Y., Buda G., Sun C., Klimstra W.B., Rossmann M.G. (2020). Cryo-EM structure of Eastern equine encephalitis virus in complex with heparan sulfate analogues. Proc. Natl. Acad. Sci. USA.

[114] Mulloy B., Gray E., Barrowcliffe T.W. (2000). Characterization of unfractionated heparin: Comparison of materials from the last 50 years. Thromb. Haemost.

[115] Aksnes I., Markussen T., Braaen S., Rimstad E. (2020). Mutation of N-glycosylation sites in salmonid alphavirus (SAV) envelope proteins attenuate the virus in cell culture. Viruses.

[116] Hikke M.C., Braaen S., Villoing S., Hodneland K., Geertsema C., Verhagen L., Frost P., Vlak J.M., Rimstad E., Pijlman G.P. (2014). Salmonid alphavirus glycoprotein E2 requires low temperature and E1 for virion formation and induction of protective immunity. Vaccine.

[117] Nelson M.A., Herrero L.J., Jeffery J.A.L., Hoehn M., Rudd P.A., Supramaniam A., Kay B.H., Ryan P.A., Mahalingam S. (2016). Role of envelope N-linked glycosylation in Ross River virus virulence and transmission. J. Gen. Virol..

[118] Knight R.L., Schultz K.L., Kent R.J., Venkatesan M., Griffin D.E. (2009). Role of N-linked glycosylation for sindbis virus infection and replication in vertebrate and invertebrate systems. J. Virol..

[119] Naim H.Y., Koblet H. (1988). Investigation of the role of glycans for the biological activity of Semliki Forest virus grown in Aedes albopictus cells using inhibitors of asparagine-linked oligosaccharides trimming. Arch. Virol..

[120] Klimstra W.B., Nangle E.M., Smith M.S., Yurochko A.D., Ryman K.D. (2003). DC-SIGN and L-SIGN can act as attachment receptors for alphaviruses and distinguish between mosquito cell- and mammalian cell-derived viruses. J. Virol..

[121] Engering A., Geijtenbeek T.B., van Vliet S.J., Wijers M., van Liempt E., Demaurex N., Lanzavecchia A., Fransen J., Figdor C.G., Piguet V. (2002). The dendritic cell-specific adhesion receptor DC-SIGN internalizes antigen for presentation to T cells. J. Immunol..

[122] Bashirova A.A., Geijtenbeek T.B., van Duijnhoven G.C., van Vliet S.J., Eilering J.B., Martin M.P., Wu L., Martin T.D., Viebig N., Knolle P.A. (2001). A dendritic cell-specific intercellular adhesion molecule 3-grabbing nonintegrin (DC-SIGN)-related protein is highly expressed on human liver sinusoidal endothelial cells and promotes HIV-1 infection. J. Exp. Med..

[123] Soilleux E.J., Morris L.S., Lee B., Pohlmann S., Trowsdale J., Doms R.W., Coleman N. (2001). Placental expression of DC-SIGN may mediate intrauterine vertical transmission of HIV. J. Pathol..

[124] Figdor C.G., van Kooyk Y., Adema G.J. (2002). C-type lectin receptors on dendritic cells and Langerhans cells. Nat. Rev. Immunol..

[125] Curtis B.M., Scharnowske S., Watson A.J. (1992). Sequence and expression of a membrane-associated C-type lectin that exhibits CD4-independent binding of human immunodeficiency virus envelope glycoprotein gp120. Proc. Natl. Acad. Sci. USA.

[126] Feinberg H., Mitchell D.A., Drickamer K., Weis W.I. (2001). Structural basis for selective recognition of oligosaccharides by DC-SIGN and DC-SIGNR. Science.

[127] Mitchell D.A., Fadden A.J., Drickamer K. (2001). A novel mechanism of carbohydrate recognition by the C-type lectins DC-SIGN and DC-SIGNR. Subunit organization and binding to multivalent ligands. J. Biol. Chem..

[128] Rice C.M., Strauss J.H. (1981). Nucleotide sequence of the 26S mRNA of Sindbis virus and deduced sequence of the encoded virus structural proteins. Proc. Natl. Acad. Sci. USA.

[129] Naim H.Y., Koblet H. (1992). Asparagine-linked oligosaccharides of Semliki Forest virus grown in mosquito cells. Arch. Virol..

[130] Gardner C.L., Burke C.W., Tesfay M.Z., Glass P.J., Klimstra W.B., Ryman K.D. (2008). Eastern and Venezuelan equine encephalitis viruses differ in their ability to infect dendritic cells and macrophages: Impact of altered cell tropism on pathogenesis. J. Virol..

[131] Crispin M., Harvey D.J., Bitto D., Bonomelli C., Edgeworth M., Scrivens J.H., Huiskonen J.T., Bowden T.A. (2014). Structural plasticity of the Semliki Forest virus glycome upon interspecies transmission. J. Proteome Res..

[132] Powell L.A., Fox J.M., Kose N., Kim A.S., Majedi M., Bombardi R., Carnahan R.H., Slaughter J.C., Morrison T.E., Diamond M.S. (2020). Human monoclonal antibodies against Ross River virus target epitopes within the E2 protein and protect against disease. PLoS Pathog..

[133] Earnest J.T., Basore K., Roy V., Bailey A.L., Wang D., Alter G., Fremont D.H., Diamond M.S. (2019). Neutralizing antibodies against Mayaro virus require Fc effector functions for protective activity. J. Exp. Med..

[134] Burke C.W., Froude J.W., Miethe S., Hulseweh B., Hust M., Glass P.J. (2018). Human-like neutralizing antibodies protect mice from aerosol exposure with Western equine encephalitis virus. Viruses.

[135] Hulseweh B., Rulker T., Pelat T., Langermann C., Frenzel A., Schirrmann T., Dubel S., Thullier P., Hust M. (2014). Human-like antibodies neutralizing Western equine encephalitis virus. MAbs.

[136] Hunt A.R., Roehrig J.T. (1985). Biochemical and biological characteristics of epitopes on the E1 glycoprotein of Western equine encephalitis virus. Virology.

[137] Broeckel R., Fox J.M., Haese N., Kreklywich C.N., Sukulpovi-Petty S., Legasse A., Smith P.P., Denton M., Corvey C., Krishnan S. (2017). Therapeutic administration of a recombinant human monoclonal antibody reduces the severity of Chikungunya virus disease in rhesus macaques. PLoS Negl. Trop. Dis..

[138] Fong R.H., Banik S.S., Mattia K., Barnes T., Tucker D., Liss N., Lu K., Selvarajah S., Srinivasan S., Mabila M. (2014). Exposure of epitope residues on the outer face of the Chikungunya virus envelope trimer determines antibody neutralizing efficacy. J. Virol..

[139] Fox J.M., Roy V., Gunn B.M., Huang L., Edeling M.A., Mack M., Fremont D.H., Doranz B.J., Johnson S., Alter G. (2019). Optimal therapeutic activity of monoclonal antibodies against Chikungunya virus requires Fc-FcγR interaction on monocytes. Sci. Immunol..

[140] Jin J., Liss N.M., Chen D.H., Liao M., Fox J.M., Shimak R.M., Fong R.H., Chafets D., Bakkour S., Keating S. (2015). Neutralizing monoclonal antibodies block Chikungunya virus entry and release by targeting an epitope critical to viral pathogenesis. Cell Rep..

[141] Kose N., Fox J.M., Sapparapu G., Bombardi R., Tennekoon R.N., de Silva A.D., Elbashir S.M., Theisen M.A., Humphris-Narayanan E., Ciaramella G. (2019). A lipid-encapsulated mRNA encoding a potently neutralizing human monoclonal antibody protects against Chikungunya infection. Sci. Immunol..

[142] Long F., Fong R.H., Austin S.K., Chen Z., Klose T., Fokine A., Liu Y., Porta J., Sapparapu G., Akahata W. (2015). Cryo-EM structures elucidate neutralizing mechanisms of anti-chikungunya human monoclonal antibodies with therapeutic activity. Proc. Natl. Acad. Sci. USA.

[143] Pal P., Dowd K.A., Brien J.D., Edeling M.A., Gorlatov S., Johnson S., Lee I., Akahata W., Nabel G.J., Richter M.K. (2013). Development of a highly protective combination monoclonal antibody therapy against Chikungunya virus. PLoS Pathog..

[144] Quiroz J.A., Malonis R.J., Thackray L.B., Cohen C.A., Pallesen J., Jangra R.K., Brown R.S., Hofmann D., Holtsberg F.W., Shulenin S. (2019). Human monoclonal antibodies against Chikungunya virus target multiple distinct epitopes in the E1 and E2 glycoproteins. PLoS Pathog..

[145] Selvarajah S., Sexton N.R., Kahle K.M., Fong R.H., Mattia K.A., Gardner J., Lu K., Liss N.M., Salvador B., Tucker D.F. (2013). A neutralizing monoclonal antibody targeting the acid-sensitive region in Chikungunya virus E2 protects from disease. PLoS Negl. Trop. Dis..

[146] Smith S.A., Silva L.A., Fox J.M., Flyak A.I., Kose N., Sapparapu G., Khomandiak S., Ashbrook A.W., Kahle K.M., Fong R.H. (2015). Isolation and characterization of broad and ultrapotent human monoclonal antibodies with therapeutic activity against Chikungunya virus. Cell Host Microbe.

[147] Agapov E.V., Razumov I.A., Frolov I.V., Kolykhalov A.A., Netesov S.V., Loktev V.B. (1994). Localization of four antigenic sites involved in Venezuelan equine encephalomyelitis virus protection. Arch. Virol..

[148] Burke C.W., Froude J.W., Rossi F., White C.E., Moyer C.L., Ennis J., Pitt M.L., Streatfield S., Jones R.M., Musiychuk K. (2019). Therapeutic monoclonal antibody treatment protects nonhuman primates from severe Venezuelan equine encephalitis virus disease after aerosol exposure. PLoS Pathog..

[149] Hunt A.R., Short W.A., Johnson A.J., Bolin R.A., Roehrig J.T. (1991). Synthetic peptides of the E2 glycoprotein of Venezuelan equine encephalomyelitis virus. II. Antibody to the amino terminus protects animals by limiting viral replication. Virology.

[150] Porta J., Jose J., Roehrig J.T., Blair C.D., Kuhn R.J., Rossmann M.G. (2014). Locking and blocking the viral landscape of an alphavirus with neutralizing antibodies. J. Virol..

[151] EnCheng S., Jing Z., Tao Y., QingYuan X., Yongli Q., WenShi W., Peng W., Liang S., Jing S., DongLai W. (2013). Analysis of murine B-cell epitopes on Eastern equine encephalitis virus glycoprotein E2. Appl. Microbiol. Biotechnol..

[152] Pereboev A.V., Razumov I.A., Svyatchenko V.A., Loktev V.B. (1996). Glycoproteins E2 of the Venezuelan and Eastern equine encephalomyelitis viruses contain multiple cross-reactive epitopes. Arch. Virol..

[153] Sun E., Zhao J., Sun L., Xu Q., Yang T., Qin Y., Wang W., Wei P., Sun J., Wu D. (2013). Comprehensive mapping of common immunodominant epitopes in the Eastern equine encephalitis virus E2 protein recognized by avian antibody responses. PLoS ONE.

[154] Zhao J., Sun E.C., Liu N.H., Yang T., Xu Q.Y., Qin Y.L., Yang Y.H., Wu D.L. (2012). Phage display identifies an Eastern equine encephalitis virus glycoprotein E2-specific B cell epitope. Vet. Immunol. Immunopathol..

[155] Zhou Q.F., Fox J.M., Earnest J.T., Ng T.S., Kim A.S., Fibriansah G., Kostyuchenko V.A., Shi J., Shu B., Diamond M.S. (2020). Structural basis of Chikungunya virus inhibition by monoclonal antibodies. Proc. Natl. Acad. Sci. USA.

[156] Mukhopadhyay S., Kuhn R.J., Rossmann M.G. (2005). A structural perspective of the flavivirus life cycle. Nat. Rev. Microbiol..

[157] Kostyuchenko V.A., Lim E.X., Zhang S., Fibriansah G., Ng T.S., Ooi J.S., Shi J., Lok S.M. (2016). Structure of the thermally stable Zika virus. Nature.

[158] Mukhopadhyay S., Kim B.S., Chipman P.R., Rossmann M.G., Kuhn R.J. (2003). Structure of West Nile virus. Science.

[159] Sirohi D., Chen Z., Sun L., Klose T., Pierson T.C., Rossmann M.G., Kuhn R.J. (2016). The 3.8 Å resolution cryo-EM structure of Zika virus. Science.

[160] Wang X., Li S.H., Zhu L., Nian Q.G., Yuan S., Gao Q., Hu Z., Ye Q., Li X.F., Xie D.Y. (2017). Near-atomic structure of Japanese encephalitis virus reveals critical determinants of virulence and stability. Nat. Commun..

[161] Fuzik T., Formanova P., Ruzek D., Yoshii K., Niedrig M., Plevka P. (2018). Structure of tick-borne encephalitis virus and its neutralization by a monoclonal antibody. Nat. Commun..

[162] Dai L., Wang Q., Qi J., Shi Y., Yan J., Gao G.F. (2016). Molecular basis of antibody-mediated neutralization and protection against flavivirus. IUBMB Life.

[163] Rey F.A., Lok S.M. (2018). Common features of enveloped viruses and implications for immunogen design for next-generation vaccines. Cell.

